# 

**DOI:** 10.1192/bjb.2024.81

**Published:** 2025-04

**Authors:** Nathan Hodson

**Affiliations:** Unit of Mental Health and Wellbeing, Warwick Medical School, University of Warwick, Coventry, UK; and Price School of Public Policy, University of Southern California, Los Angeles, USA. Email: Nathan.Hodson@warwick.ac.uk

**Figure:**
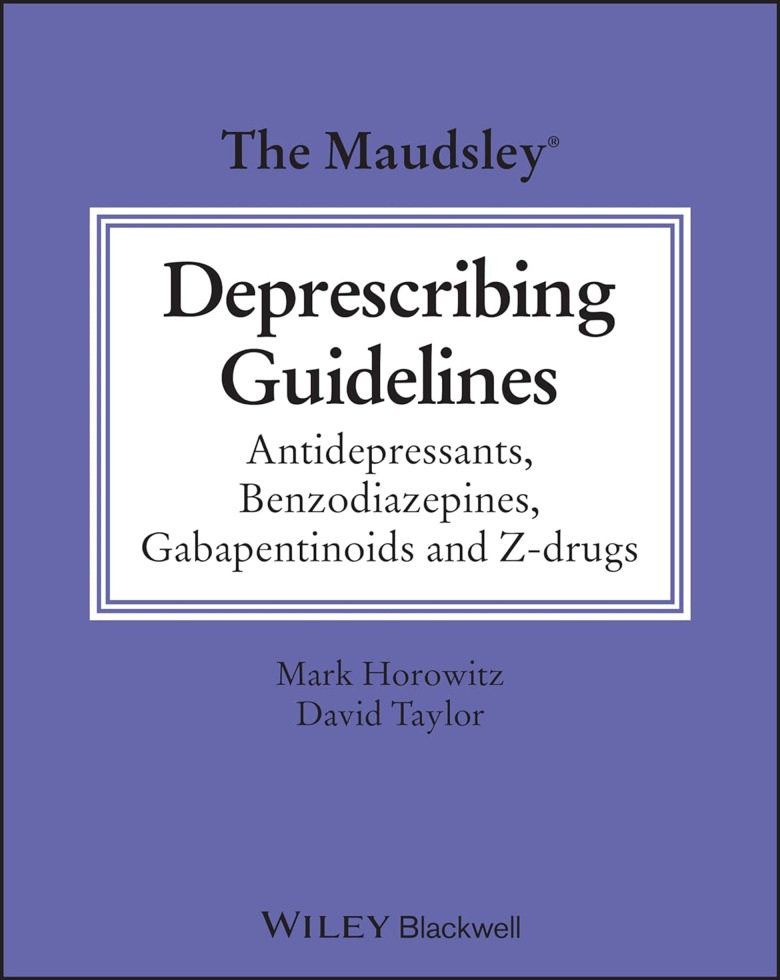

Over the last 25 years the Maudsley Prescribing Guidelines (MPG) have provided authoritative and practical advice to inform the clinical use of psychopharmacological interventions. For the first time, in 2024, they have published *deprescribing* guidelines – a handy reference applying the same detailed but user-friendly approach to the process of stopping medications safely.

The deprescribing guidelines are divided into four sections. The first comprises a series of short essays making the case for stopping psychiatric medication and describing the clinical and pharmacodynamic pitfalls. Key concepts from this section – distinguishing withdrawal symptoms from rebound symptoms and considering receptor occupancy rather than dosage alone – are applied in the subsequent sections which contain detailed guidance for stopping antidepressants, benzodiazepines, Z-drugs, and gabapentinoids. Sections two to four open by summarizing the probable withdrawal effects before two or three pages are dedicated to each drug within each class.

The best way to use this book is to read section one on the general principles, but then refer to the specific pages for each drug when clinically needed. The tapering tables and receptor occupancy graphs for each drug are one of the greatest strengths of this text. By contrast, the section opening texts overviewing the side-effects of antidepressants or gabapentinoids are too broad-brush, commenting on classes in general rather than specific drugs. Tables showing the frequency of adverse effects of ‘antidepressants’ raise more questions (Which drugs? Which doses? When?) than they answer.

The other major limitation of the book is that antipsychotics and mood stabilizers are notable by their absence. The authors touch on the importance of safely discontinuing antipsychotics in passing, and perhaps intend to include them in a future volume.

This book is part of a wider project aiming to reduce harmful and unnecessary prescribing and it is likely to make significant progress towards that aim. The authors report that clinicians avoid deprescribing due to fear of withdrawal effects and rebound symptoms, and this book directly addresses those concerns. However, the impact of this book may be limited because, although clear about *how* to deprescribe medications, it lacks specific guidance on *when* to stop prescriptions. Further work along these lines may also include evidence-based insights about motivating patients to engage with the deprescribing process. Specific education, prompts and nudges regarding the circumstances under which deprescribing is appropriate would make it more likely that this book will have the system-wide impact to which Taylor and Horowitz aspire.

